# Which factors contribute most to genome size variation within angiosperms?

**DOI:** 10.1002/ece3.7222

**Published:** 2021-01-31

**Authors:** Dandan Wang, Zeyu Zheng, Ying Li, Hongyin Hu, Zhenyue Wang, Xin Du, Shangzhe Zhang, Mingjia Zhu, Longwei Dong, Guangpeng Ren, Yongzhi Yang

**Affiliations:** ^1^ State Key Laboratory of Grassland Agro‐Ecosystem Institute of Innovation Ecology & School of Life Sciences Lanzhou University Lanzhou China

**Keywords:** angiosperm, genome size, long terminal repeat, polyploidization, repeat sequences

## Abstract

Genome size varies greatly across the flowering plants and has played an important role in shaping their evolution. It has been reported that many factors correlate with the variation in genome size, but few studies have systematically explored this at the genomic level. Here, we scan genomic information for 74 species from 74 families in 38 orders covering the major groups of angiosperms (the taxonomic information was acquired from the latest Angiosperm Phylogeny Group (APG IV) system) to evaluate the correlation between genome size variation and different genome characteristics: polyploidization, different types of repeat sequence content, and the dynamics of long terminal repeat retrotransposons (LTRs). Surprisingly, we found that polyploidization shows no significant correlation with genome size, while LTR content demonstrates a significantly positive correlation. This may be due to genome instability after polyploidization, and since LTRs occupy most of the genome content, it may directly result in most of the genome variation. We found that the LTR insertion time is significantly negatively correlated with genome size, which may reflect the competition between insertion and deletion of LTRs in each genome, and that the old insertions are usually easy to recognize and eliminate. We also noticed that most of the LTR burst occurred within the last 3 million years, a timeframe consistent with the violent climate fluctuations in the Pleistocene. Our findings enhance our understanding of genome size evolution within angiosperms, and our methods offer immediate implications for corresponding research in other datasets.

## Graphical abstract

Genome size is a biodiversity trait that shows staggering diversity across the flowering plants, which plays an important role in shaping the evolution of plants. Here, we scan the genomic information of 54 species from 52 families covering the major groups of angiosperms and evaluate the correlation between variations of genome sizes and different genome characters. We surprisingly find only LTRs significantly positively correlate with genome size variation, while polyploidization does not, and genome size acts as an evolutionary character that helps each species adapt to different environments with different patterns (different types of TEs burst or polyploidization).

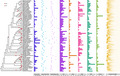

## 1 INTRODUCTION

Genome size (also known as the C‐value) refers to the total amount of DNA contained within one copy of a single complete genome; it is broadly constant within an organism (Greilhuber et al., 2005; Swift, 1950). More and more species’ genome sizes have been assessed since early studies in the 1950s, covering more than 12,273 land plants, 6,222 animals, and 2,353 fungi (Gregory, 2015; Kullman et al., 2005; Pellicer & Leitch, 2020). Researchers have also discovered that eukaryotic genome size varies greatly over more than a 100,000‐fold range and fails to correlate well with apparent complexity; this is the well‐known ‘‘C‐value paradox’’ (Eddy, 2012; Thomas Jr, 1971). Among the most widely studied land plants, angiosperms (10,770 species searched) exhibit an astonishing diversity of genome size, with a maximum variation by a factor of approximately 2,440 (Leitch et al., 2019; Pellicer et al., 2018), that is, the smallest angiosperm plant genome reported so far is *Genlisea tuberosa* (Lentibulariaceae, 61 Mb/1C) (Fleischmann et al., 2014), a carnivorous angiosperm endemic to Brazil, and the largest is *Paris japonica* (Pellicer et al., 2010), a monocot lily species in the Melanthiaceae family with an astonishingly large genome made up of ca. 149,000 Mb/1C of DNA. Furthermore, the dramatic variation in genome size can occur even among congeners. For example, the variation in genome size can reach ~30‐fold in Brassicaceae (0.16–4.63 Gb), ~37‐fold in Rosaceae (0.10–3.57 Gb), and ~44‐fold in Asteraceae (0.39–25.60 Gb) (Leitch et al., 2019).

Several mechanisms have been proposed to account for the variation in genome size, such as recombination rate, tandem repeats (Tiley & Burleigh, 2015), transposable elements (TEs), and polyploidization, but the relative contribution of these different mechanisms seems to vary between species (Bennetzen et al., 2005). Polyploidization can directly increase the genome size by doubling all the genome contents, and this occurs widely within angiosperms. With the exception of *Amborella*, nearly all the angiosperms have undergone polyploidization events, and the different major lineages (i.e., Ceratophyta, eudicots, monocots, magnolia, and Nymphaeales) have all experienced independent polyploidization events (Initiative, 2019; Van de Peer et al., 2017; Yang et al., 2020). Repeated DNA sequences account for the majority of the genomic DNA in most plant species, occurring in a few to millions of copies. The content of repeated sequences shifts significantly across plant genomes. It can be as low as ~3%, for example, in *Utricularia gibba* (Ibarra‐Laclette et al., 2013), and is as high as ~85% in *Zea mays* (Schnable et al., 2009). Among the repetitive sequences, tandem repeats usually occupy a small proportion of the genome and the main repeats fall into four types of transposable elements (TEs): long terminal repeat elements (LTRs), long interspersed nuclear elements (LINEs), short interspersed nuclear elements (SINEs) and DNA transposon repeat elements (DNA transposons). Among the different types of TEs, LTRs usually occupy the largest proportion of plant genomes and dynamic bursts have acted as major contributors to the genome size differences between plants (Lee & Kim, 2014).

With the development of high‐throughput sequencing technologies, more and more angiosperm genomes have been sequenced, assembled, and made publicly available (https://www.plabipd.de/), providing an opportunity to investigate the variation in genome size within angiosperms systematically. Here, we scan the genome sizes of 74 flowering plant genomes from 74 families covering 38 orders (taxonomic foundation sourced from APG IV) and evaluate the correlation between the genome size and three factors: polyploidization, the proportion of repetitive elements, and LTR activity. Based on a series of correlation analyses, we explore which factor is mainly responsible for the genome size variation in angiosperms.

## 2 MATERIALS AND METHODS

### 2.1 Genome datasets collection

In this study, we sampled 74 species, the genomes of which were derived from previous research, in 74 families representing 38 orders. This dataset included genomes from NCBI, Ensembl Plants, and many other individual genome databases, such as the Herbal Medicine Omics Database, gigaDB datasets, and the *Panax notoginseng* Genome Database. The 74 plant genomes were sampled from 38 diverse orders of five main taxa among the angiosperms. Detailed information about these 74 species and their data sources is presented in Table S1.

### 2.2 Repeat sequence identification

To check whether certain types of repeat sequence or whole‐genome duplications may have caused the variation in angiosperm genomes, we examined the duplicated genes of 74 species separately and identified whole‐genome duplication events from published literature (Table S3). The different kinds of duplicated genes were identified using different pipelines. Tandem repeats, which include minisatellites, microsatellites, and others, divided by nucleotide length were identified using Tandem Repeats Finder v4.90 (Benson, 1999), while transposable elements were identified using RepeatMasker and RepeatModeler. The TEs were identified using a combination of Repbase (Bao et al., 2015) and the de novo prediction results of RepeatModeler. We then used perl script to calculate the proportion of different types of repetitive elements in the genome. The entire pipeline was deposited at Github (https://github.com/dandanWang2019/genome_size_pipeline).

### 2.3 Polyploidization fold assessment

The polyploidization fold (PF) was calculated by the formula: *PF = 2^m^* × *3^n^*, where *m* refers to the number of times of the whole‐genome duplication events and *n* refers to the number of times of the triplication events**,** with data sourced from the literature. Because of common duplications, ancient polyploidization events in angiosperms were not taken into account (Soltis et al., 2003).

### 2.4 LTR insertion date calculation

Insertion dates were calculated following the methods in the published literature (SanMiguel et al., 1998). The downloaded genomes were scanned using LTR_Finder (Xu & Wang, 2007), and full‐length LTRs were extracted by perl scripts. LTR 5′ and 3′ pairs were aligned with MUSCLE (Edgar, 2004) and ClustalW2 (Larking et al., 2007), and the divergence between LTR pairs was calculated in PHYLIP v3.696. The insertion time of each LTR was estimated in millions of years using the formula: *T = K/2r* (*r* = 1.3 × 10^–8^ per site and per year) (Ma & Bennetzen, 2004), where *K* refers to nucleotide substitution rates and the arithmetic mean of insertion time was calculated for each species in millions of years.

### 2.5 Correlation analysis

Nuclear genome size estimates were determined through scripts from the downloaded genome file and scaled by ancestral haploid genome size of angiosperms (1.73 pg × 978 Mb/pg = 1691.94 Mb) (Carta et al., 2020). The regressions were performed on the proportions of repetitive elements, polyploidization fold, and mean LTR insertion time against genome size fold. The correlation between potentially related factors and genome size fold was calculated using the R lme4 package (Bates et al., 2015). To consider the possible roles of divergence time in the relationship between LTR abundance and insertion time, we conducted a multiple regression with age and insertion time as predictors of abundance (Figure 3h and Table S4). We also analyzed the associations between the factors and genome size fold in a phylogenetic context. The phylogenetic tree was acquired from a recent angiosperm phylogeny study (Li et al., 2019) and pruned with Newick Utilities v1.6.0 (Junier & Zdobnov, 2010). The fitting of a PGLS model in a phylogenetic context with Brownian motion was conducted using the gls function from the nlme package (Pinheiro et al., 2017). All correlation analysis results and phylogenetic trees used for PGLS analysis are presented in the supplementary files (Table S5 and Figure S3). Results were considered significant when *p* < .05.

## 3 RESULTS

### 3.1 Genome collection and repeat sequences identification

**Figure 1:**
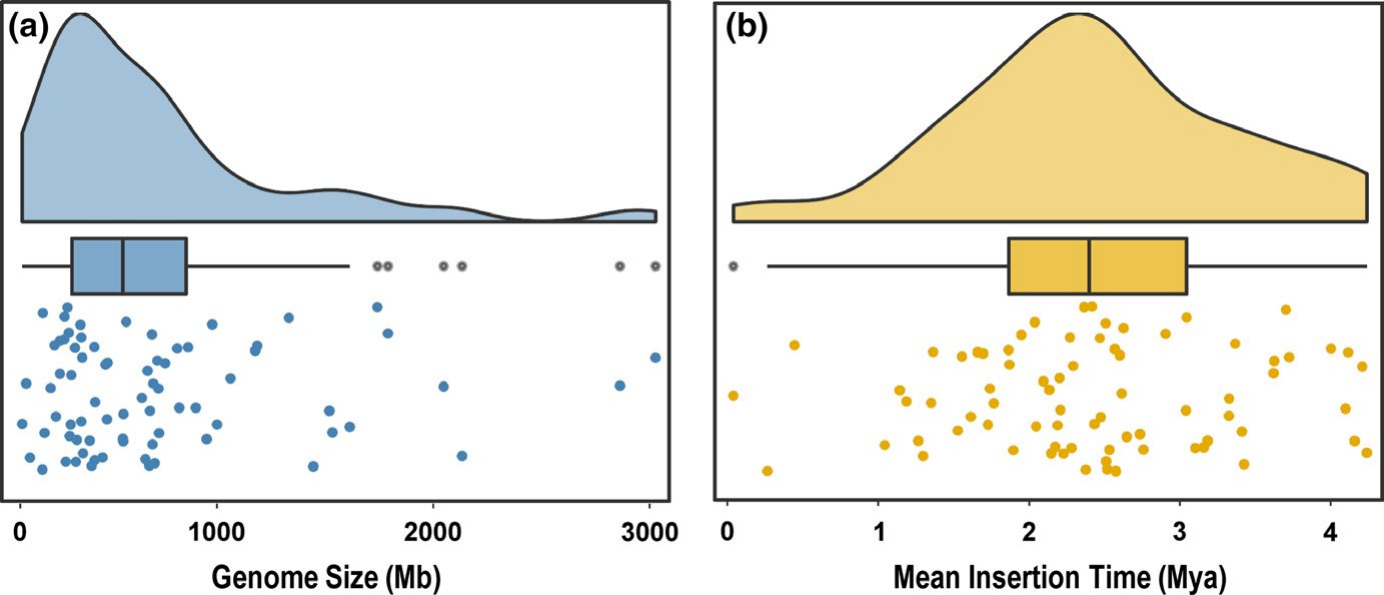
The distributions of plant genome sizes (a) and mean LTR insertion times (b). The density curves represent the distribution, while the scatter diagram and the box‐plot show the statistics including median, quartile and the outliers

**Figure 2:**
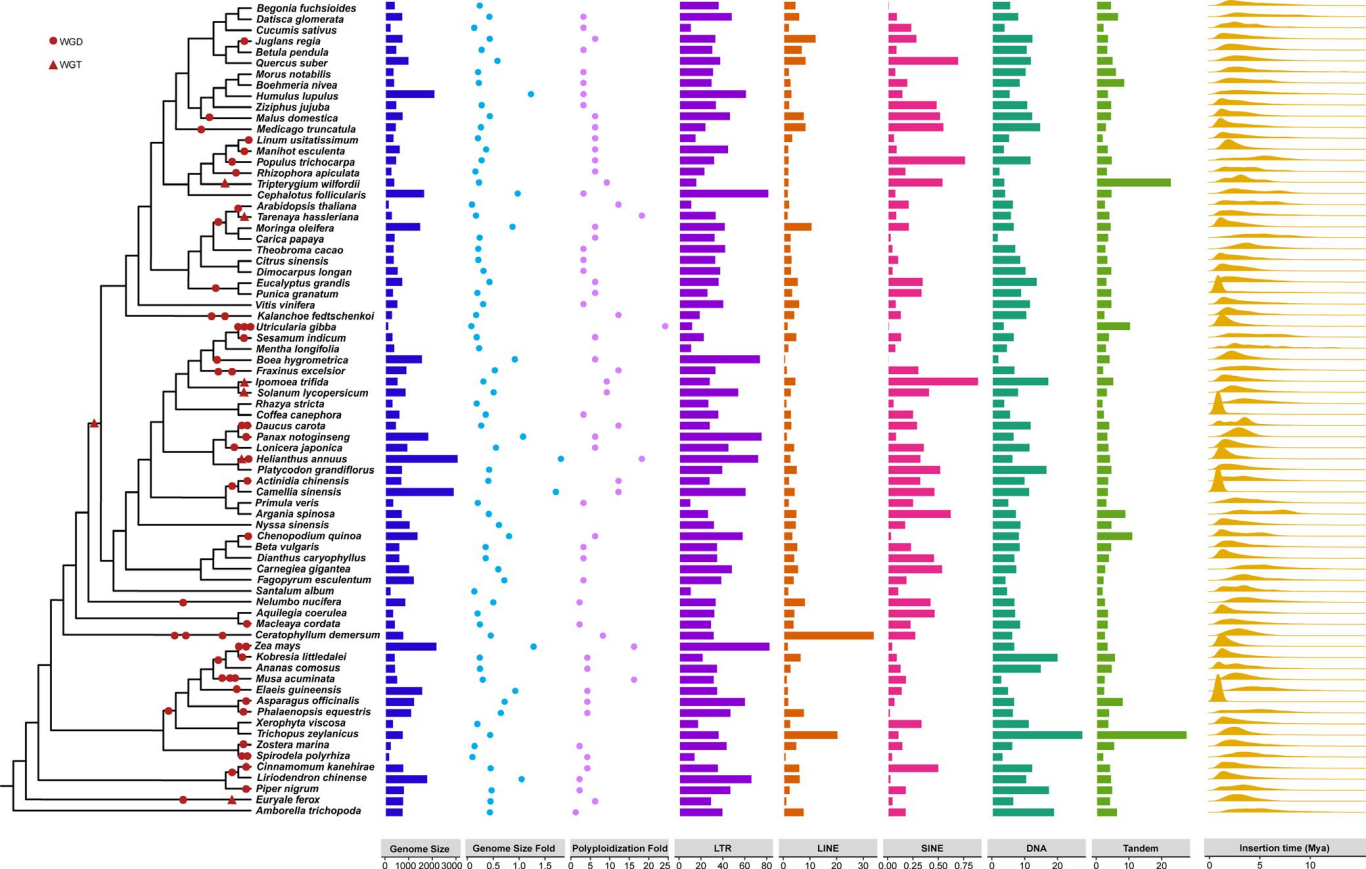
Representation of phylogeny and the correlation factors analyzed in 74 genomes. GF: genome size fold, PF: polyploidization fold, tandem: tandem repeats, and LTR insertion dates. GF indicates the genome size fold in plants scaled by the ancestral genome size for angiosperms and PF indicates the value of polyploidization fold which is the number of times that whole‐genome duplication and the whole‐genome triplication occurred. LTR‐tandem indicates different proportions of corresponding repeating elements in genomes as a percentage (%). Mean insertion date indicates the estimated distribution of LTR insertion dates in plants in millions of years. The WGDs and WGTs are labeled in the branches. The topology information cited is from Li et al. (2019)

The genome assemblies of 74 flowering plant genomes from 74 families of 38 orders were collected from the NCBI Genome database, GigaDB, and other specific databases (Table S1). Our selected genomes covered the major groups of angiosperms, including two basal angiosperms, twelve monocots, three magnolias, one Ceratophyllale, and 56 eudicots (Table S1). A 30‐fold variation in genome size was detected within these 74 species ranging from ~100 Mb (*U. gibba*) to ~ 3,027 Mb (*Helianthus annuus*), with an average of 730.0 Mb and a median value of 566.9 Mb; a genome size of over 1,000 Mb was identified in more than 15 species (Figure 1a and Table S2). A standard method was adopted to annotate the repeat sequences within each genome, and we found that repeats make up a large proportion of the genome in all species ranging from 21.59% in *Spirodela polyrhiza* to 83.23% in *Z. mays* (Table S2). *U. gibba* was estimated to contain 31.81% repeats and the result is higher than in a previous study (Ibarra‐Laclette et al., 2013). This may be attributed to the growing number of recognizable repeats and the integration of different types of software in this analysis (Table S2). Transposable elements were the major components of repeats rather than tandem repeats, and the four main types of TEs varied within different species. LTRs were the dominant TE type in the 72 species, ranging from 9.49% to 81.76%, and the two species with the most abundant LTRs were *Z. mays* (81.76%) and *Cephalotus follicularis* (80.61%). Surprisingly, in *Ceratophyllum demersum*, LINEs were the dominant components, accounting for 33.60% of the genome, while LTRs accounted for 30.76%. In *Tripterygium wilfordii*, tandem repeats accounted for 22.54% of the genome, exceeding the 14.88% of LTRs. The two species with the most abundant DNA transposons were *Trichopus zeylanicus* (27.29%) and *Kobresia littledalei* (19.71%), while SINEs contributed very little to the genome size in any of the species (≤0.88%) (Figures 2 and S1; Table S2).

### 3.2 LTRs insertion time

As the main component of repeats, we further explored the LTR activity in relation to LTR insertion time. We found a large proportion of the estimated mean LTR insertions occurred recently, with an average insertion time of 2.42 million years ago (Mya) and a median value of 2.40 Mya (Figure 1b). *Elaeis guineensis*, *Argania spinose*, *Carica papaya*, *A. trichopoda*, *Carnegiea gigantean*, and *Populus trichocarpa* had mean LTR insertion times greater than 4 Mya (4.0–4.24 Mya), and all contained a small proportion of LTRs (25.57%–47.41%). In contrast, the younger LTR insertions occurred typically in species that had a relatively high percentage of LTRs, such as *Asparagus officinalis* (59.26%; mean LTR insertion time: 0.04 Mya) and *Camellia sinensis* (59.87%; mean LTR insertion time: 0.26 Mya) (Figures 2 and S2).

### 3.3 Polyploidization event characteristics

As complex and uncertain polyploidization events occurred within ancestral seed plants and angiosperms over a very long time (>200 Mya) (Van de Peer et al., 2017), it is difficult to confirm the real polyploizidization fold (PF) of each species. Here, we assumed the polyploidization fold of *A. trichopoda* to be 1, experiencing only ancient polyploidization events that occurred in the ancestral angiosperms. We collected information on all polyploidization events reported in published papers for each species and scaled the number in relation to *A. trichopoda* (Table S3). Within our dataset, all the species had a value larger than 1, as all the angiosperms except for *A. trichopoda* have experienced additional WGD events. *U. gibba*, with the smallest genome size, had the highest polyploidization value of 24 (Figure 2 and Table S3).

### 3.4 Correlation between the factors analyzed and genome size

**Figure 3:**
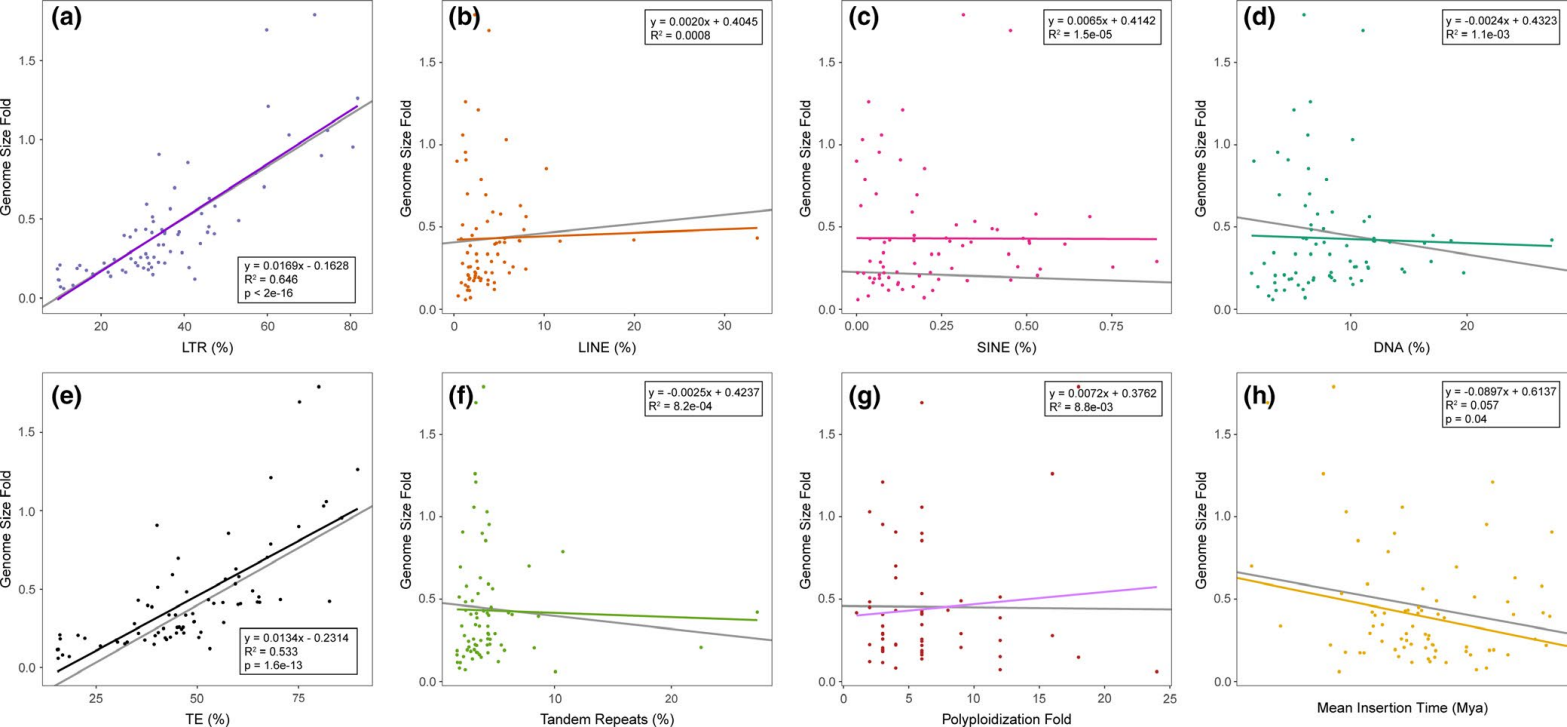
Different factors (in colors) as a function of genome size. Different factors fitted against genome size fold (genome size scaled by ancestral genome size for angiosperms). The gray lines represent the estimated result from phylogenetic least squares (PGLS) analysis. (a‐f) The relationship between the proportion of different repeated elements against genome size fold in 74 species. (g) The absence of a relationship between polyploidization fold and genome size fold. (h) The mean date of LTR insertions was significantly correlated with genome size fold. Lines are plots of linear regressions

A series of factors including the proportion of repetitive elements, polyploidization fold, and mean LTR insertion time were examined in the correlation analysis. To ensure phylogenetic independence, we also constructed phylogenetic generalized least‐square models (PGLS) to fit the data and the results remained similar, with the exception of the mean LTR insertion time (Figure 3). Across the five repeat elements examined, a strong significant positive correlation was only observed between genome size and the proportion of LTR elements (Figure 3). Species with larger genomes had a relatively larger proportion of LTRs. This was evidenced by the linear regression comparing the proportion of LTR against genome size fold (*R* ^2^ = 0.646, *y* = 0.0169*x*–0.1628, *p* < 2.0 × 10^–16^; Figure 3a).

With respect to LTR activity, we also considered the effects of divergence times in the relationship between LTR abundance and insertion time. The results of the linear regression showed that LTR insertion time was negatively correlated with genome size fold (*R* ^2^ = 0.056, *y* = −0.08581*x* + 0.4080, *p* = .04; Figure 3h). Species with large genomes consistently have relatively recent mean LTR insertion times; for example, *Asparagus officinalis* and *Camellia sinensis*, with the youngest mean LTR insertion times, had comparatively larger genomes, whereas LTR insertions generally occurred earlier in species with smaller genomes like *Amborella trichopoda*, *C. papaya* and *P. trichocarpa* (Figure 2 and Table S2). Nevertheless, when taking account of phylogenetic nonindependence, the correlation was no longer observed. In addition, to investigate whether polyploidy, as a crucial driving force, also affected the genome size holistically, we calculated the correlation between polyploidization fold and genome size fold. We found that polyploidization fold was not related to the variation in genome size fold (*R* ^2^ = 0.0088; Figure 3g).

## 4 DISCUSSION

### 4.1 Absence of a relationship between polyploidization and genome size

From our broad perspective, we were surprised to find that genome size is not significantly correlated to polyploidization, even though the latter is widely known to increase genomes by the inheritance of an additional set (or sets) of chromosomes (Bruggmann et al., 2006; Iorizzo et al., 2016). Multiple ancient polyploidy events occurred in plants around 100 to 120 million years ago and after that relatively recent WGDs occurred in many lineages during the evolution of angiosperms (Fawcett & Van de Peer, 2010; Wu et al., 2020). Those polyploidization helped flowering plants improved acclimatization during severe environmental changes and survival until now (Fawcett & Van de Peer, 2010; Zhang et al., 2020). While, a larger genome comes with the ecological burden of needing more macronutrients to build nucleic acids, particularly nitrogen and phosphorus, with the latter being limited in numerous natural environments (Šmarda et al., 2013; Vitousek et al., 2010). This cost usually varies greatly at different stages as environment changes. For example, CO2 has often been considered to have a dominant role in plant survival as the potentially limiting photosynthetic resource (Boyce & Zwieniecki, 2012), and the atmospheric CO_2_ concentrations have fluctuated greatly over the past 400 million years (Rothman, 2002). The CO_2_ content in the atmosphere during 100–120 million years ago was much higher, and in the last few million years, it showed a significantly decline (Boyce & Zwieniecki, 2012; Foster et al., 2017), which resulting in an increase in the cost to angiosperms of recent polyploidization (Rothman, 2002). In other words, polyploidization expands genome size in a short period accompanying greater environmental pressure and nutritional needs and maintaining a large genome usually collapsed when external resources in the environment become tense. Thus, diploidization usually follows polyploidization, especially within angiosperms (Dodsworth et al., 2016; Meudt et al., 2015). Diploidization involves the removal of extra DNA (often repetitive DNA) and extraneous gene copies and occurs through recombination‐based deletion and other mechanisms, while retaining duplicated genes, some of which may have new or altered functions (Adams & Wendel, 2005; Dodsworth et al., 2016). Diploidization can also downsize the genome by chromosome number reduction, which potentially involves complex chromosomal rearrangements (including fusions and fissions) (Dodsworth et al., 2016; Franzke et al., 2011; Meudt et al., 2015). Diploidization is considered the key to the evolutionary success of angiosperms, and it has resulted in irregular genome reduction, which explains why polyploidization did not exhibit a significant positive linear correlation with genome size.

### 4.2 Effects of TEs especially LTRs on genome size variation

TEs accounted for the most genome content and contributed the most to the genome size variation (Figure 3e). Previous studies have attributed the bigger genome to long‐term amplification of TEs, which is associated with a naturally occurring reduction in the efficiency of symmetric DNA methylation in *Arabis alpine* (Willing et al., 2015), and the reduced quantity of small RNAs associated with TE silencing in *Picea abies* (gymnosperms) (Nystedt et al., 2013). In our study, we also found the TEs, especially LTRs exhibited the most significant positive correlation with the genome size variation (Figure 3a, e). So, another reason may be that polyploidization could also induce the activity and burst of TEs, which further diluted the influence of a linear correlation between polyploidization and genome size. Polyploidization usually causes chromatin modifications and epigenetic regulation to accumulate more TEs and produce a bigger genome (McClintock, 1984; Springer et al., 2016; Vicient & Casacuberta, 2017). For example, a widespread DNA methylation variation in TEs was observed in autotetraploid rice and was accompanied by changes in the abundance of 24‐nucleotide small interfering RNAs (siRNAs) (Zhang et al., 2015), and demethylation of TEs has been observed in newly formed allopolyploids (Parisod et al., 2009; Yaakov & Kashkush, 2011).

Besides polyploidization, many other variables could also lead to TE bursts and cause changes in genome size; these include abiotic stress, domestication, and the mating system changes (Belyayev, 2014). In a natural population, stress‐induced bursts of TEs, especially driven by environmental changes, are important and of special interest because this phenomenon may underlie micro‐ and macro‐evolutionary events and ultimately support the generation and maintenance of biological diversity. We found a burst of LTR insertions mainly in comparatively recent times (<3 Mya, Figure S2 and Table S2), which is likely to have increased plant resistance to the violently fluctuating climate during the Early Pleistocene cooling (Hofreiter & Stewart, 2009; Xu et al., 2018). We also found that the mean insertion time showed a slightly negative correlation with genome size variations (Figure 3h), which is different from previous studies (Nystedt et al., 2013; Willing et al., 2015). This weak negative correlation may be caused by the competition between TE insertion and elimination. Plant genomes have experienced multiple rounds of TE outbreaks in their evolutionary histories, leading abundant TE families to escape from silencing mechanisms (El Baidouri & Panaud, 2013; Fultz et al., 2015; Lisch & Slotkin, 2011). However, as the genome tends to be stable, most TEs are eliminated and only some TEs are able to combat this with silencing, by inactivating the systems that have evolved to recognize them (Fu et al., 2013; McCue et al., 2013). So the ancient TEs usually account for a small proportion of the genome and the recent TEs are mainly responsible for the genome size (Divashuk et al., 2020; Oliver et al., 2013).

### 4.3 Adaptation of flowering plants to the environment through genome size variation

Genome size is generally considered to be an evolutionary character, indicating that any change is not a random event, but usually a response to external environmental fluctuations (artificial or natural) (Levin, 2002; Pellicer et al., 2018). Whole‐genome duplications and LTR insertions increase the biological complexity and size of the genome, generating novel functions, and altering gene expression patterns. This allows plants to adapt to the environment more easily (Oliver et al., 2013, Van de Peer et al., 2017). Thanks to the polyploidization that was closely associated with complicated climate changes, plants have survived for a long time even in the face of the severe environmental conditions, while retaining certain gene duplicates (Cai et al., 2019; Wu et al., 2020). The insertion of LTRs has been concentrated in the last million years when there have been drastic global environmental changes, indicating their important role in plant survival. This potentially accounts for the extreme diversity in angiosperms compared with the sister clade, gymnosperms, with low LTR activity, but abundant TEs (Kovach et al., 2010; Oliver et al., 2013).

We also found that, when faced with similar environmental conditions, plants may respond in different ways. Within the aquatic plants *Ceratophyllum demersum* and *Euryale ferox*, the proportion of repeats differs greatly: LINE and LTR are the dominant TE types, respectively. In the carnivorous plants *C. follicularis* and *U. gibba*, not only does the proportion of the repetitive elements vary greatly, but this is also the case for the frequency of whole‐genome duplication events. *C. follicularis*, with a high proportion of repeated elements, experienced a round of WGT, while *U. gibba* with a low proportion experienced three rounds of whole‐genome duplication events and a whole‐genome triplication event. Apparently, adaption through different TEs and polyploidization has helped the angiosperms to develop unique modus vivendi, resulting in the survival of a range of taxa. In spite of the fact that diverse strategies may be adopted among species, they still have to confront the same circumstances.

In summary, we systematically scanned 74 species belonging to 74 families from 38 orders, covering the major groups of angiosperms. We performed correlation analysis to compare genome size and polyploidization, different repeat content and LTR insertion times. Our results have enhanced our understanding of genome size variation within angiosperms, and our pipeline will also be of use in future studies examining genome size evolution.

## CONFLICT OF INTEREST

The authors declare that they have no conflict of interest.

## AUTHOR CONTRIBUTION

**Dandan Wang:** Conceptualization (equal); Data curation (equal); Formal analysis (equal); Investigation (equal); Methodology (equal); Validation (equal); Visualization (lead); Writing‐original draft (equal). **Zeyu Zheng:** Data curation (equal); Formal analysis (equal); Methodology (supporting). **Ying Li:** Data curation (equal); Formal analysis (equal); Writing‐review & editing (equal). **Hongyin Hu:** Data curation (equal); Formal analysis (equal). **Zhenyue Wang:** Data curation (equal); Investigation (equal); Methodology (equal). **Xin Du:** Data curation (equal); Methodology (equal). **Shangzhe Zhang:** Data curation (equal); Formal analysis (equal); Investigation (equal). **Mingjia Zhu:** Data curation (equal); Methodology (equal). **Longwei Dong:** Conceptualization (equal); Methodology (equal); Software (equal). **Guangpeng Ren:** Conceptualization (equal); Funding acquisition (equal); Investigation (equal); Methodology (equal); Resources (equal); Writing‐review & editing (equal). **Yongzhi Yang:** Conceptualization (equal); Funding acquisition (equal); Investigation (supporting); Methodology (supporting); Project administration (equal); Resources (equal); Writing‐original draft (supporting); Writing‐review & editing (supporting).

## Supporting information

Supplementary Material

## Data Availability

All the repeat elements annotation and LTR sequence insertion times are available from figshare (https://doi.org/10.6084/m9.figshare.12514085.v3).
