# Supplementary material for: Which factors contribute most to genome size variation within angiosperms?
Source: Ecol Evol. 2021 Jan 31;11(6):2660–8. doi: 10.1002/ece3.7222 (PMC7981209; doi:10.1002/ece3.7222)
Supplement: Supplementary file 1 — Supplementary Material [file ECE3-11-2660-s001.docx]

**Supplementary Materials**

**Figure S1. The proportions of different repetitive elements (in colors).** LTR accounts for the largest percentage among most species except for *Ceratophyllum demersum* (33.60% in LINE) while SINE minimum in all repeats.

**
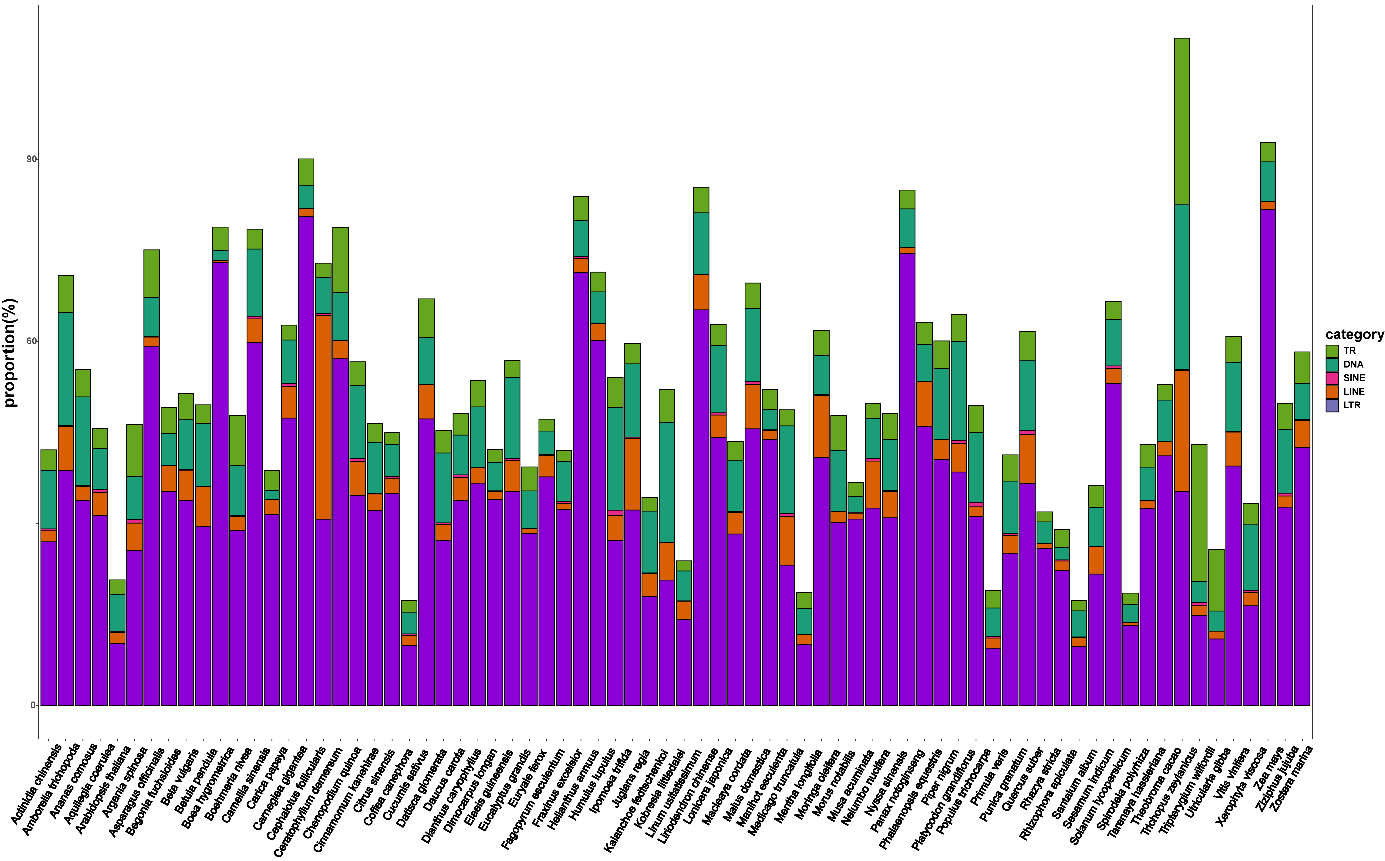
**

**Figure S2.** Density curve show the distribution of LTRs insertion date in 74 species. Most species have a peak in LTRs insertion while others inserted in several periods.


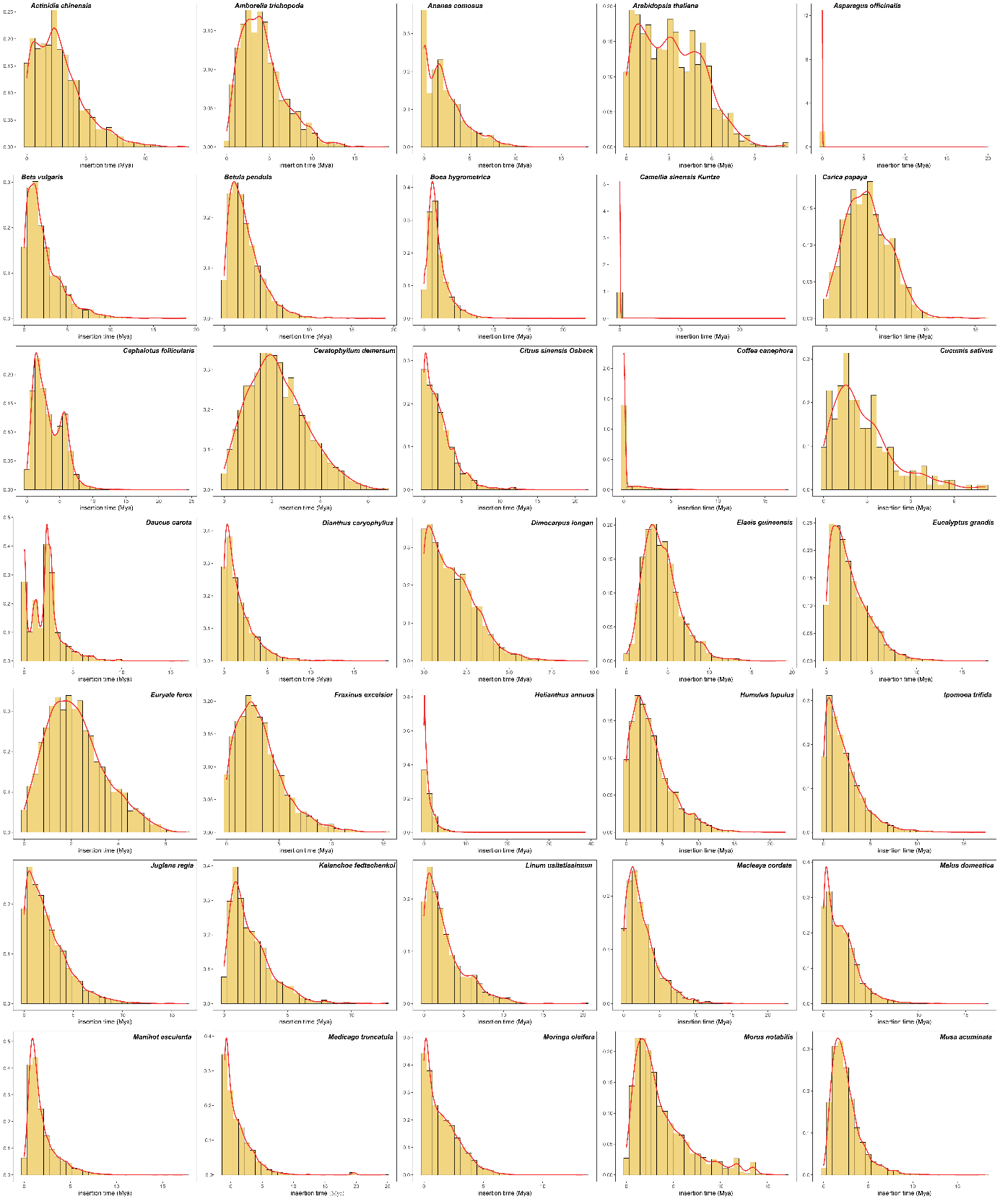


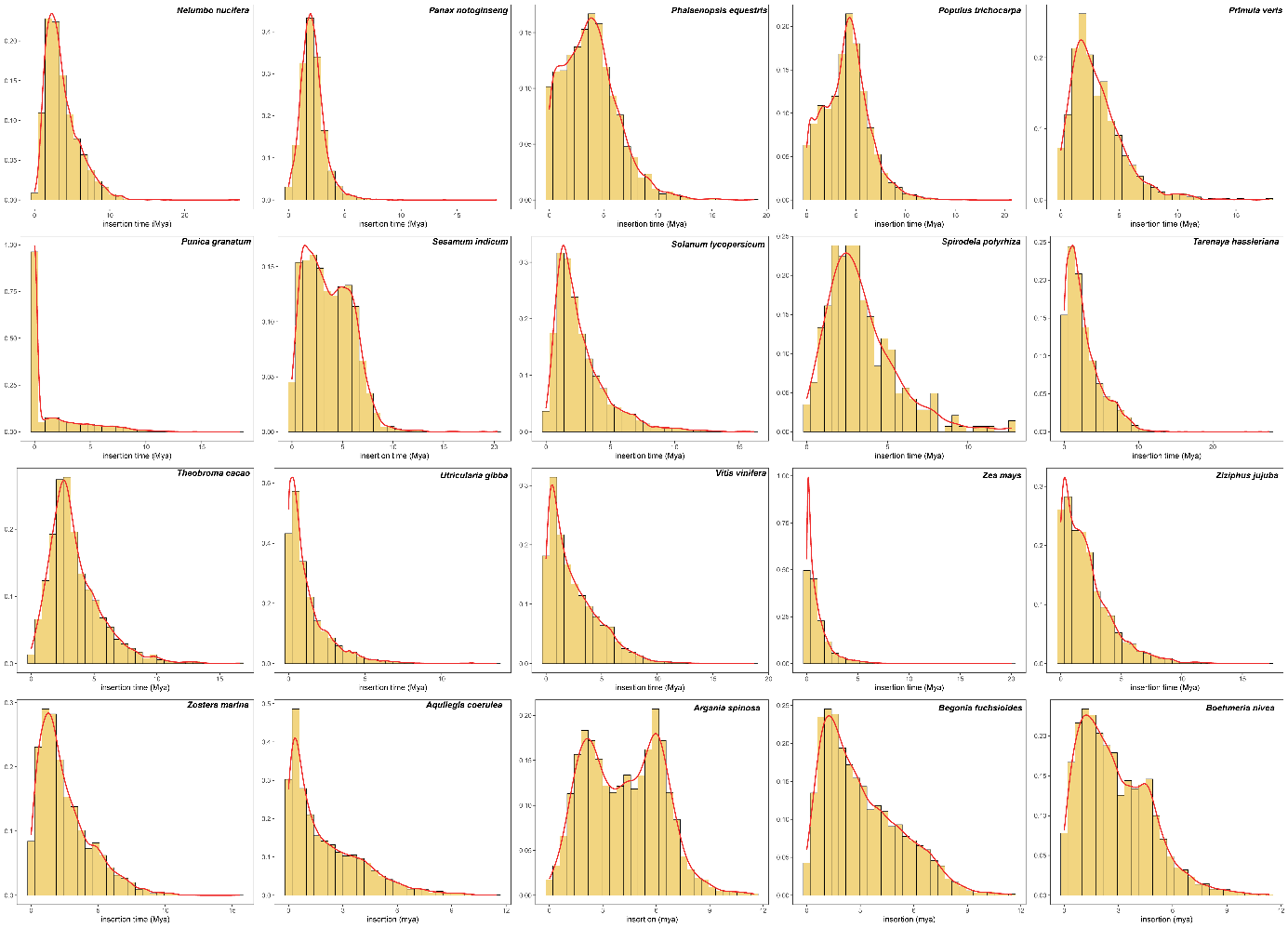


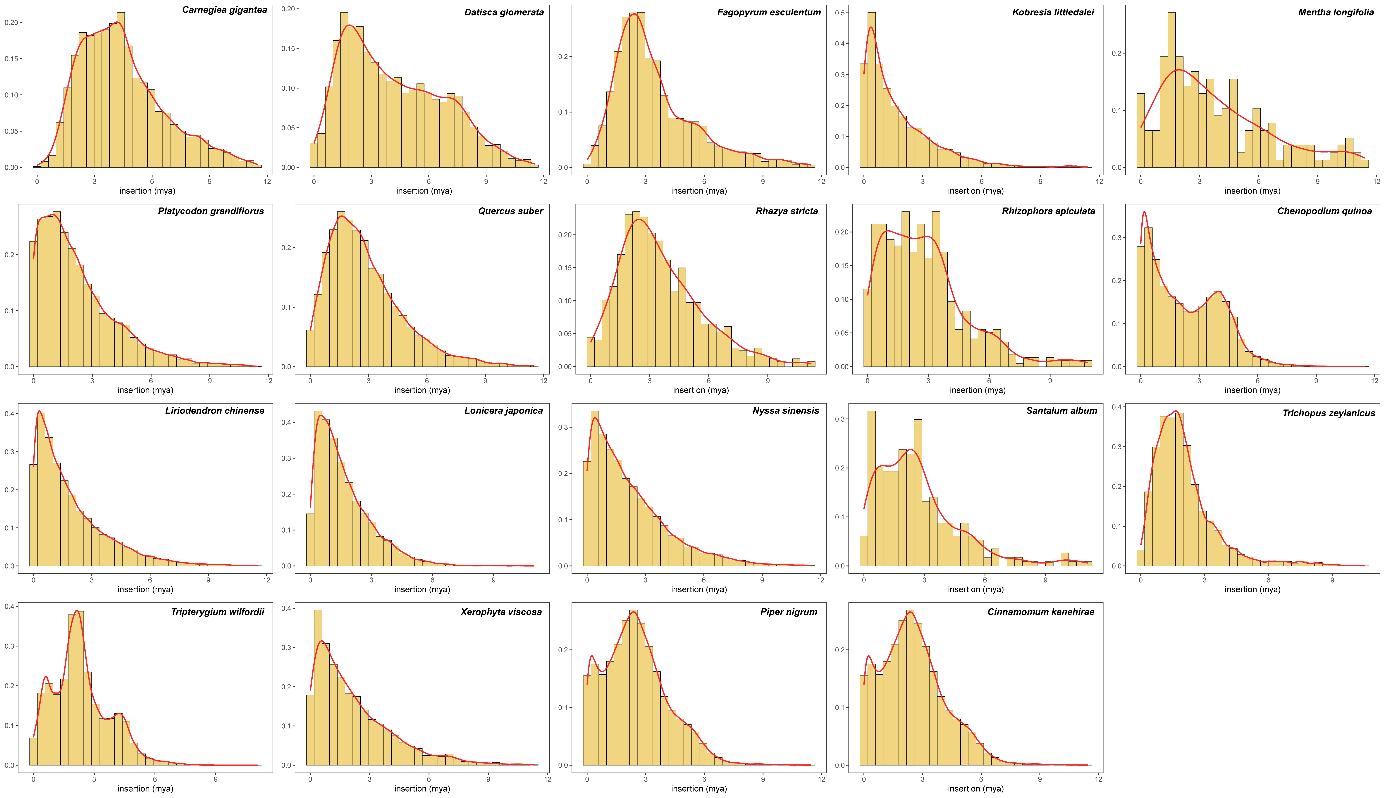


**Figure S3.** Phylogenetic tree of the 73 species in the phylogenetic generalized least-square models (PGLS) analysis dataset.


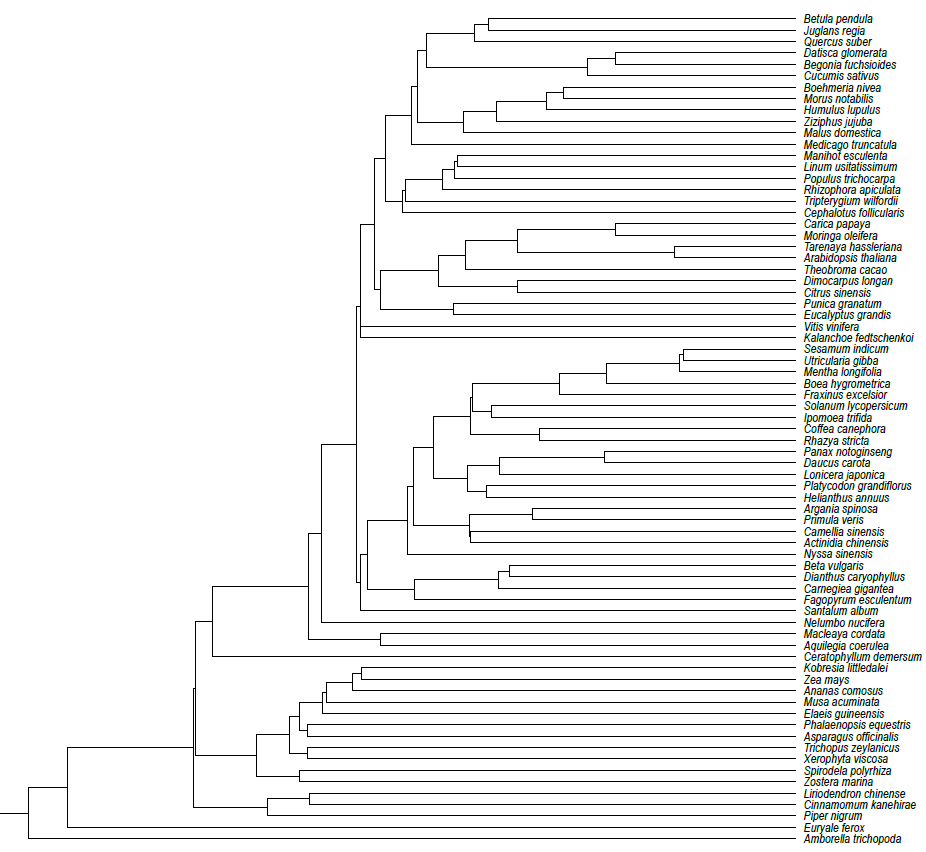


**Table S1.** Basic information on 74 plants sampled including families, orders, source, version and GenBank accession.

| Species | Family | Orders | Database | Version | Genbank accession | Ploidy |
| --- | --- | --- | --- | --- | --- | --- |
| *Actinidia chinensis* | Actinidiaceae | Ericales | NCBI | Kiwifruit_v1 | GCA_000467755.1 | Diploid |
| *Amborella trichopoda* | Amborellaceae | Amborellales | NCBI | AMTR1.0 | GCA_000471905.1 | Diploid |
| *Ananas comosus* | Bromeliaceae | Poales | NCBI | ASM154086v1 | GCA_001540865.1 | Diploid |
| *Arabidopsis thaliana* | Brassicaceae | Brassicales | NCBI | TAIR10.1 | GCA_000001735.2 | Diploid |
| *Asparagus officinalis* | Asparagaceae | Asparagales | NCBI | Aspof.V1 | GCA_001876935.1 | Diploid |
| *Beta vulgaris* | Amaranthaceae | Caryophyllales | NCBI | RefBeet-1.2.2 | GCA_000511025.2 | Diploid |
| *Betula pendula* | Betulaceae | Fagales | NCBI | Bpev01 | GCA_900184695.1 | Diploid |
| *Boea hygrometrica* | Gesneriaceae | Lamiales | NCBI | Boea_hygrometrica.v1 | GCA_001598015.1 | Diploid |
| *Camellia sinensis* Kuntze | Theaceae | Ericales | NCBI | AHAU_CSS_2 | GCA_004153795.2 | Diploid |
| *Carica papaya* | Caricaceae | Brassicales | NCBI | ASM131004v1 | GCA_001310045.1 | Diploid |
| *Cephalotus follicularis* | Cephalotaceae | Oxalidales | NCBI | Cfol_1.0 | GCA_001972305.1 | Diploid |
| *Ceratophyllum demersum* | Ceratophyllaceae | Ceratophyllales | CoGe(https://genomevolution.org/CoGe/GenomeInfo.pl?gid=56572) | Ceratophyllum demersum | - | Diploid |
| *Cinnamomum kanehirae* | Lauraceae | Laurales | NCBI | ASBRC_Ckan_1.0 | GCA_003546025.1 | Diploid |
| *Citrus sinensis* Osbeck | Rutaceae | Sapindales | NCBI | Csi_valencia_1.0 | GCA_000317415.1 | Diploidz |
| *Coffea canephora* | Rubiaceae | Gentianales | NCBI | AUK_PRJEB4211_v1 | GCA_900059795.1 | Diploid |
| *Cucumis sativus* | Cucurbitaceae | Cucurbitales | NCBI | ASM407v2 | GCA_000004075.2 | Diploid |
| *Daucus carota* | Apiaceae | Apiales | NCBI | ASM162521v1 | GCA_001625215.1 | Diploid |
| *Dianthus caryophyllus* | Caryophyllaceae | Caryophyllales | NCBI | DCA_r1.0 | GCA_000512335.1 | Diploid |
| *Dimocarpus longan* | Sapindaceae | Sapindales | gigaDB (http://dx.doi.org/10.5524/100276) | Erigeron breviscapus | - | Diploid |
| *Elaeis guineensis* | Arecaceae | Arecales | NCBI | EG5 | GCA_000442705.1 | Diploid |
| *Eucalyptus grandis* | Myrtaceae | Myrtales | NCBI | Egrandis1_0 | GCA_000612305.1 | Diploid |
| *Euryale ferox* | Nymphaeaceae | Nymphaeales | CoGe(https://genomevolution.org/CoGe/GenomeInfo.pl?gid=56574) | Euryale ferox | - | Diploid |
| *Fraxinus excelsior* | Oleaceae | Lamiales | NCBI | BATG-0.5 | GCA_900149125.1 | Diploid |
| *Helianthus annuus* | Asteraceae | Asterales | NCBI | HanXRQr1.0 | GCA_002127325.1 | Diploid |
| *Humulus lupulus* | Cannabaceae | Rosales | NCBI | hl_SW_version_1.0.fasta | GCA_000831365.1 | Diploid |
| *Ipomoea trifida* | Convolvulaceae | Solanales | NCBI | ASM357666v1 | GCA_003576665.1 | Diploid |
| *Juglans regia* | Juglandaceae | Fagales | NCBI | wgs.5d | GCA_001411555.1 | Diploid |
| *Kalanchoe fedtschenkoi* | Crassulaceae | Saxifragales | NCBI | K_fedtschenkoi_M2_v1 | GCA_002312845.1 | Diploid |
| *Linum usitatissimum* | Linaceae | Malpighiales | NCBI | ASM22429v2 | GCA_000224295.2 | Diploid |
| *Macleaya cordata* | Papaveraceae | Ranunculales | NCBI | MC_HNAU_1.0 | GCA_002174775.1 | Diploid |
| *Malus domestica* | Rosaceae | Rosales | NCBI | ASM211411v1 | GCA_002114115.1 | Diploid |
| *Manihot esculenta* | Euphorbiaceae | Malpighiales | NCBI | Manihot esculenta v6 | GCA_001659605.1 | Diploid |
| *Medicago truncatula* | Fabaceae | Fabales | NCBI | MedtrA17_4.0 | GCA_000219495.2 | Diploid |
| *Moringa oleifera* | Moringaceae | Brassicales | Herbal Medicine Omics Database (ftp://202.203.187.112:2222/genome/dendrobe/) | Moringa oleifera | - | Diploid |
| *Morus notabilis* | Moraceae | Rosales | NCBI | ASM41409v2 | GCA_000414095.2 | Diploid |
| *Musa acuminata* | Musaceae | Zingiberales | NCBI | ASM31385v2 | GCA_000313855.2 | Diploid |
| *Nelumbo nucifera* | Nelumbonaceae | Proteales | NCBI | ASM303368v1 | GCA_003033685.1 | Diploid |
| *Zea mays* | Poaceae | Poales | NCBI | B73 RefGen_v4 | GCA_000005005.6 | Diploid |
| *Panax notoginseng* | Araliaceae | Apiales | Panax notoginseng (http://www.plantkingdomgdb.com/panax_notoginseng/data/) | Panax notoginseng | - | Diploid |
| *Phalaenopsis equestris* | Orchidaceae | Asparagales | NCBI | ASM126359v1 | GCA_001263595.1 | Diploid |
| *Populus trichocarpa* | Salicaceae | Malpighiales | NCBI | Pop_tri_v3 | GCA_000002775.3 | Diploid |
| *Primula veris* | Primulaceae | Ericales | NCBI | ASM78844v1 | GCA_000788445.1 | Diploid |
| *Punica granatum* | Lythraceae | Myrtales | NCBI | ASM220158v1 | GCA_002201585.1 | Diploid |
| *Sesamum indicum* | Pedaliaceae | Lamiales | NCBI | S_indicum_v1.0 | GCA_000512975.1 | Diploid |
| *Solanum lycopersicum* | Solanaceae | Solanales | NCBI | SL3.0 | GCA_000188115.3 | Diploid |
| *Spirodela polyrhiza* | Araceae | Alismatales | NCBI | ASM198140v1 | GCA_001981405.1 | Diploid |
| *Tarenaya hassleriana* | Cleomaceae | Brassicales | NCBI | ASM46358v1 | GCA_000463585.1 | Diploid |
| *Theobroma cacao* | Malvaceae | Malvales | NCBI | Criollo_cocoa_genome_V2 | GCA_000208745.2 | Diploid |
| *Utricularia gibba* | Lentibulariaceae | Lamiales | NCBI | U_gibba_v2 | GCA_002189035.1 | Diploid |
| *Vitis vinifera* | Vitaceae | Vitales | NCBI | 12X | GCA_000003745.2 | Diploid |
| *Ziziphus jujuba* | Rhamnaceae | Rosales | NCBI | ZizJuj_1.1 | GCA_000826755.1 | Diploid |
| *Zostera marina* | Zosteraceae | Alismatales | NCBI | Zosma_marina.v.2.1 | GCA_001185155.1 | Diploid |
| *Aquilegia coerulea* | Ranunculaceae | Ranunculales | NCBI | Aquilegia_coerulea_v1 | GCA_002738505.1 | Diploid |
| *Argania spinosa* | Sapotaceae | Ericales | NCBI | arg_spin_01 | GCA_003260245.1 | Diploid |
| *Begonia fuchsioides* | Begoniaceae | Cucurbitales | NCBI | ASM325500v1 | GCA_003255005.1 | Diploid |
| *Boehmeria nivea* | Urticaceae | Rosales | NCBI | ASM293701v1 | GCA_002937015.1 | Diploid |
| *Carnegiea gigantea* | Cactaceae | Caryophyllales | NCBI | SGP5_Cgig_v1.3 | GCA_002740515.1 | Diploid |
| *Chenopodium quinoa* | Amaranthaceae | Caryophyllales | NCBI | ASM168347v1 | GCF_001683475.1 | Tetraploid |
| *Datisca glomerata* | Datiscaceae | Cucurbitales | NCBI | ASM325502v1 | GCA_003255025.1 | Diploid |
| *Fagopyrum esculentum* | Polygonaceae | Caryophyllales | NCBI | FES_r1.0 | GCA_001661195.1 | Diploid |
| *Kobresia littledalei* | Cyperaceae | Poales | NCBI | ASM1111435v1 | GCA_011114355.1 | Diploid |
| *Liriodendron chinense* | Magnoliaceae | Magnoliales | NCBI | NJFU_Lchi_2.0 | GCA_003013855.2 | Diploid |
| *Lonicera japonica* | Caprifoliaceae | Dipsacales | https://bigd.big.ac.cn/search/?dbId=gwh&q=SAMC097356 | GWHAAZE00000000 | - | Diploid |
| *Mentha longifolia* | Lamiaceae | Lamiales | NCBI | Mlong1.0 | GCA_001642375.1 | Hexaploid |
| *Nyssa sinensis* | Nyssaceae | Cornales | NCBI | ASM863837v1 | GCA_008638375.1 | Diploid |
| *Piper nigrum* | Piperaceae | Piperales | http://cotton.hzau.edu.cn/EN/download.php | Piper nigrum genome | - | Diploid |
| *Platycodon grandiflorus* | Campanulaceae | Asterales | NCBI | RDA_PgrJ_v1 | GCA_004681165.1 | Diploid |
| *Quercus sube* | Fagaceae | Fagales | NCBI | CorkOak1.0 | GCA_002906115.1 | Diploid |
| *Rhazya stricta* | Apocynaceae | Gentianales | NCBI | RHA1.0 | GCA_001752375.1 | Diploid |
| *Rhizophora apiculata* | Rhizophoraceae | Malpighiales | NCBI | Rap_scaffold_v2 | GCA_900174605.1 | Diploid |
| *Santalum album* | Santalaceae | Santalales | NCBI | ASM291163v1 | GCA_002911635.1 | Diploid |
| *Trichopus zeylanicus* | Dioscoreaceae | Dioscoreales | CoGe(https://genomevolution.org/coge/GenomeInfo.pl?gid=54631) | v01 | - | Diploid |
| *Tripterygium wilfordii* | Celastraceae | Celastrales | NCBI | ASM1340144v1 | GCA_013401445.1 | Diploid |
| *Xerophyta viscosa* | Velloziaceae | Pandanales | http://www.bioinformatics.nl/xerophyta/portal/data/ | Xviscosa | - | Octoploid |

**Table S2** Statistics for the factors analyzed including genome size, polyploidization fold, proportions of all repeat sequences, divergence time and mean LTRs insertion time for species sampled.

| Species | Genome size (Mb) | Polyploidization fold | LTR (%) | LINE (%) | SINE (%) | DNA (%) | Tandem repeats (%) | Transposable elements  (%) | Mean LTR insertion date (Mya) | Divergence age (Mya) |
| --- | --- | --- | --- | --- | --- | --- | --- | --- | --- | --- |
| *Actinidia chinensis* | 653.93 | 12 | 27.0128 | 1.8087 | 0.3118 | 9.6597 | 3.37 | 38.793 | 2.735084 | 86.39 |
| *Amborella trichopoda* | 706.33 | 1 | 38.8032 | 7.2468 | 0.1688 | 18.6230 | 6.07 | 64.8418 | 4.117630 | 208.75 |
| *Ananas comosus* | 381.90 | 4 | 33.8178 | 2.3352 | 0.1188 | 14.6244 | 4.47 | 50.8962 | 2.271333 | 120.51 |
| *Aquilegia coerulea* | 301.98 | - | 31.3773 | 3.7859 | 0.4532 | 6.7821 | 3.33 | 42.3985 | 1.351808 | 87.39 |
| *Arabidopsis thaliana* | 119.67 | 12 | 10.2516 | 1.8319 | 0.1987 | 6.0815 | 2.37 | 18.3637 | 3.185040 | 32.79 |
| *Argania spinosa* | 670.10 | - | 25.5694 | 4.5379 | 0.6123 | 7.0203 | 8.63 | 37.7399 | 4.211308 | 71.38 |
| *Asparagus officinalis* | 1187.54 | 4 | 59.2620 | 1.4974 | 0.0578 | 6.4708 | 7.83 | 67.288 | 0.038816 | 41.28 |
| *Begonia fuchsioides* | 373.91 | - | 35.3316 | 4.2266 | 0.0032 | 5.2858 | 4.25 | 44.8472 | 2.577423 | 48.95 |
| *Beta vulgaris* | 566.18 | 3 | 33.8493 | 4.8612 | 0.2206 | 8.2038 | 4.30 | 47.1349 | 2.292841 | 77.7 |
| *Betula pendula* | 435.91 | 3 | 29.5396 | 6.5536 | 0.0786 | 10.3300 | 3.08 | 46.5018 | 2.509227 | 57.36 |
| *Boea hygrometrica* | 1521.36 | 6 | 73.0459 | 0.3420 | 0.0001 | 1.6838 | 3.82 | 75.0718 | 2.038283 | 47.75 |
| *Boehmeria nivea* | 344.62 | 3 | 28.8406 | 2.3718 | 0.1846 | 8.1860 | 8.27 | 39.583 | 2.475115 | 62.96 |
| *Camellia sinensis Kuntze* | 2863.25 | 12 | 59.8650 | 3.8815 | 0.4524 | 11.0568 | 3.28 | 75.2557 | 0.263842 | 72.5 |
| *Carica papaya* | 369.78 | 6 | 31.5508 | 2.4017 | 0.0209 | 1.4853 | 3.34 | 35.4587 | 4.160652 | 48.8 |
| *Carnegiea gigantea* | 980.35 | - | 47.4137 | 5.1758 | 0.5271 | 7.1533 | 2.47 | 60.2699 | 4.100865 | 28.53 |
| *Cephalotus follicularis* | 1614.52 | 3 | 80.6082 | 1.2860 | 0.0666 | 3.7273 | 4.41 | 85.6881 | 3.327617 | 79.32 |
| *Ceratophyllum demersum* | 733.26 | 8 | 30.7579 | 33.6005 | 0.2631 | 5.9190 | 2.40 | 70.5405 | 2.227444 | 158.62 |
| *Chenopodium quinoa* | 1333.55 | 6 | 57.1767 | 3.0179 | 0.0249 | 7.9221 | 10.72 | 68.1416 | 2.418194 | - |
| *Cinnamomum kanehirae* | 730.42 | 4 | 34.6432 | 5.6319 | 0.4895 | 11.9992 | 3.92 | 52.7638 | 2.418194 | 100.5 |
| *Citrus sinensis Osbeck* | 327.67 | 3 | 32.1453 | 2.7585 | 0.0946 | 8.3512 | 3.14 | 43.3496 | 2.04792 | 68.75 |
| *Coffea canephora* | 568.61 | 3 | 35.0141 | 2.4996 | 0.2414 | 5.2523 | 2.07 | 43.0074 | 0.443807 | 69.7 |
| *Cucumis sativus* | 193.83 | 3 | 9.8870 | 1.7250 | 0.2237 | 3.5889 | 1.94 | 15.4246 | 2.760129 | 56.57 |
| *Datisca glomerata* | 688.40 | 3 | 47.2669 | 5.6106 | 0.0819  0 | 7.7208 | 6.37 | 60.6802 | 3.703981 | 48.95 |
| *Daucus carota* | 421.50 | 12 | 27.1991 | 2.6532 | 0.2799 | 11.5161 | 3.70 | 41.6483 | 2.207974 | 51.87 |
| *Dianthus caryophyllus* | 567.66 | 3 | 33.8572 | 3.7290 | 0.4471 | 6.5684 | 3.58 | 44.6017 | 1.741477 | 77.7 |
| *Dimocarpus longan* | 495.33 | 3 | 36.6937 | 2.5555 | 0.0394 | 9.9620 | 4.31 | 49.2506 | 1.695271 | 75.5 |
| *Elaeis guineensis* | 1535.02 | 4 | 33.9532 | 1.3362 | 0.1290 | 4.6224 | 2.15 | 40.0408 | 4.240392 | 96.17 |
| *Eucalyptus grandis* | 691.27 | 6 | 35.3153 | 5.0645 | 0.3365 | 13.3568 | 2.85 | 54.0731 | 2.648203 | 83.6 |
| *Euryale ferox* | 725.23 | 6 | 28.3573 | 0.8076 | 0.0395 | 6.1958 | 3.94 | 35.4002 | 2.188386 | 114 |
| *Fagopyrum esculentum* | 1177.69 | 3 | 37.7132 | 3.5301 | 0.1776 | 3.8606 | 1.95 | 45.2815 | 2.905385 | 77.72 |
| *Fraxinus excelsior* | 867.46 | 12 | 32.3716 | 0.9624 | 0.2931 | 6.5881 | 1.85 | 45.2815 | 3.158758 | 46.39 |
| *Helianthus annuus* | 3027.84 | 18 | 71.4129 | 2.3100 | 0.3140 | 5.9867 | 3.93 | 80.0236 | 1.186500 | 75.29 |
| *Humulus lupulus* | 2049.21 | 3 | 60.2295 | 2.7014 | 0.1355 | 5.1251 | 3.27 | 68.1915 | 3.412017 | 67.71 |
| *Ipomoea trifida* | 492.38 | 9 | 27.1879 | 4.1634 | 0.8824 | 16.9133 | 4.92 | 49.147 | 2.203793 | 82.53 |
| *Juglans regia* | 700.62 | 6 | 32.2471 | 11.7588 | 0.2742 | 12.0523 | 3.32 | 56.3324 | 2.366294 | 80.3 |
| *Kalanchoe fedtschenkoi* | 256.35 | 12 | 18.0138 | 3.7572 | 0.1210 | 10.1881 | 2.22 | 32.0801 | 2.096861 | 96.19 |
| *Kobresia littledalei* | 373.85 | 4 | 20.7299 | 6.137 | 0.0806 | 19.7091 | 5.45 | 46.6566 | 1.142538 | 78.85 |
| *Linum usitatissimum* | 316.17 | 6 | 14.2324 | 3.0002 | 0.0526 | 4.9268 | 1.67 | 22.212 | 2.614895 | 86.92 |
| *Liriodendron chinense* | 1742.42 | 2 | 65.2387 | 5.7951 | 0.0176 | 10.1678 | 4.23 | 81.2192 | 1.363296 | 113 |
| *Lonicera japonica* | 903.81 | 6 | 44.1966 | 3.7126 | 0.3477 | 11.1585 | 3.46 | 59.4154 | 1.297385 | 68.99 |
| *Macleaya cordata* | 377.83 | 2 | 28.3056 | 3.5241 | 0.2170 | 8.3200 | 3.22 | 40.3667 | 2.568216 | 112.13 |
| *Malus domestica* | 702.96 | 6 | 45.6309 | 7.3001 | 0.5070 | 11.9717 | 4.25 | 65.4097 | 1.896602 | 90.25 |
| *Manihot esculenta* | 582.12 | 6 | 43.9018 | 1.5040 | 0.0790 | 3.3894 | 3.25 | 48.8742 | 1.868480 | 60.23 |
| *Medicago truncatula* | 412.80 | 6 | 23.1864 | 7.9682 | 0.5398 | 14.4172 | 2.65 | 46.1116 | 1.863664 | 84.16 |
| *Mentha longifolia* | 353.29 | 3 | 10.1134 | 1.5672 | 0.0670 | 4.2938 | 2.68 | 16.0414 | 3.045654 | 30.14 |
| *Moringa oleifera* | 1447.00 | 6 | 40.8954 | 10.2401 | 0.1999 | 6.3722 | 4.12 | 57.7076 | 1.529478 | 48.8 |
| *Morus notabilis* | 320.38 | 3 | 30.2481 | 1.7587 | 0.0673 | 9.9985 | 5.72 | 42.0726 | 3.367169 | 62.96 |
| *Musa acuminata* | 472.23 | 16 | 30.7795 | 0.9557 | 0.1707 | 2.5663 | 2.30 | 34.4722 | 2.519430 | 54.13 |
| *Nelumbo nucifera* | 817.27 | 2 | 32.5772 | 7.7584 | 0.4134 | 6.6142 | 2.39 | 47.3632 | 3.622106 | 83.42 |
| *Nyssa sinensis* | 1001.45 | - | 30.9752 | 4.3096 | 0.1635 | 8.4140 | 4.35 | 43.8623 | 1.660365 | 50.52 |
| *Panax notoginseng* | 1790.76 | 6 | 74.5124 | 0.9786 | 0.0734 | 6.3163 | 3.11 | 81.8807 | 2.147853 | 51.87 |
| *Phalaenopsis equestris* | 1064.05 | 4 | 46.0523 | 7.3709 | 0.0127 | 6.1011 | 3.66 | 59.537 | 3.725017 | 127.02 |
| *Piper nigrum* | 761.22 | 2 | 45.9508 | 2.0155 | 0.1695 | 17.0789 | 4.58 | 65.2147 | 2.376404 | 67.89 |
| *Platycodon grandiflorus 680.177651 38.5254 4.7027 0.5083 16.3444 4.37 15.31 1.766846 81.19 60.0808* | 680.18 | - | 38.5254 | 4.7027 | 0.5083 | 16.3444 | 4.37 | 60.0808 | 1.766846 | 81.19 |
| *Populus trichocarpa* | 434.13 | 6 | 31.1591 | 1.6247 | 0.7531 | 11.4886 | 4.49 | 45.0255 | 4.004179 | 58.03 |
| *Primula veris* | 309.69 | 3 | 9.4923 | 1.6738 | 0.2412 | 4.7110 | 2.84 | 16.1183 | 3.040841 | 71.38 |
| *Punica granatum* | 296.38 | 6 | 25.1467 | 2.9424 | 0.3251 | 8.6153 | 4.31 | 37.0295 | 1.555563 | 70.23 |
| *Quercus suber* | 953.30 | - | 36.6633 | 7.9894 | 0.6855 | 11.6114 | 4.71 | 56.9496 | 2.435038 | 87.36 |
| *Rhazya stricta* | 274.35 | - | 25.9334 | 0.8256 | 0.0488 | 3.4799 | 1.66 | 30.2877 | 3.102365 | 55.28 |
| *Rhizophora apiculata* | 232.06 | 6 | 22.2991 | 1.5682 | 0.1664 | 2.0295 | 2.98 | 26.0632 | 2.511962 | 65.78 |
| *Santalum album* | 196.10 | - | 9.7310 | 1.507 | 0.094 | 4.3100 | 1.70 | 15.642 | 2.280923 | 79.89 |
| *Sesamum indicum* | 274.91 | 6 | 21.7045 | 4.4802 | 0.1227 | 6.3670 | 3.59 | 32.6744 | 3.625511 | 30.34 |
| *Solanum lycopersicum* | 827.75 | 9 | 53.1309 | 2.4732 | 0.3985 | 7.6647 | 2.97 | 63.6673 | 2.626015 | 82.53 |
| *Spirodela polyrhiza* | 136.67 | 4 | 13.2391 | 0.4777 | 0.0345 | 2.9580 | 1.87 | 16.7093 | 3.325219 | 130.57 |
| *Tarenaya hassleriana* | 249.93 | 18 | 32.4750 | 1.2766 | 0.0759 | 5.4973 | 3.73 | 39.3248 | 2.601262 | 32.79 |
| *Theobroma cacao* | 324.72 | 3 | 41.1864 | 2.3244 | 0.0386 | 6.8234 | 2.54 | 50.3728 | 3.426346 | 76.77 |
| *Trichopus zeylanicus* | 713.41 | - | 35.3483 | 19.9350 | 0.0976 | 27.2885 | 27.35 | 82.6694 | 1.726750 | 110.94 |
| *Tripterygium wilfordii* | 348.53 | 9 | 14.8779 | 1.6026 | 0.5316 | 3.5149 | 22.54 | 20.527 | 2.173462 | 111.53 |
| *Utricularia gibba* | 100.69 | 24 | 11.0205 | 1.2831 | 0.0038 | 3.3086 | 10.08 | 15.616 | 1.264790 | 30.07 |
| *Vitis vinifera* | 485.33 | 3 | 39.4987 | 5.6077 | 0.0702 | 11.3409 | 4.36 | 56.5175 | 2.470758 | 118.21 |
| *Xerophyta viscosa* | 295.46 | - | 16.4820 | 2.1909 | 0.3254 | 10.9287 | 3.43 | 29.927 | 1.612500 | 115.91 |
| *Ziziphus jujuba* | 437.75 | 3 | 32.7360 | 1.829 | 0.4738 | 10.4757 | 4.29 | 45.5145 | 2.135168 | 74.82 |
| *Zostera marina* | 203.91 | 2 | 42.5747 | 4.4823 | 0.1352 | 5.9149 | 5.20 | 53.1071 | 2.534073 | 51.75 |
| *Zea mays* | 2134.37 | 16 | 81.7570 | 1.2911 | 0.0356 | 6.5389 | 3.21 | 89.6226 | 1.042637 | 75.99 |

**Table S3.** Times of the whole genome duplications (WGDs) and the whole genome triplications (WGT) in all species sampled were sourced from the literature.

| Species | WGDs | WGTs | Genome fold | Reference |
| --- | --- | --- | --- | --- |
| *Actinidia chinensis* | 2 | 1 | 12 | Qiao et al. 2019 |
| *Amborella trichopoda* | 0 | 0 | 1 | Qiao et al. 2019 |
| *Ananas comosus* | 2 | 0 | 4 | Qiao et al. 2019 |
| *Asparagus officinalis* | 2 | 0 | 4 | Van de Peer et al. 2017 |
| *Arabidopsis thaliana* | 2 | 1 | 12 | Qiao et al. 2019 |
| *Beta vulgaris* | 0 | 1 | 3 | Qiao et al. 2019 |
| *Betula pendula* | 0 | 1 | 3 | Salojärvi et al. 2017 |
| *Boea hygrometrica* | 1 | 1 | 6 | Qiao et al. 2019 |
| *Boehmeria nivea* | 0 | 1 | 3 | Ren et al. 2018 |
| *Camellia sinensis* | 1 | 1 | 6 | Wei et al. 2018 |
| *Carica papaya* | 1 | 1 | 6 | Qiao et al. 2019 |
| *Cephalotus follicularis* | 0 | 1 | 3 | Fukushima et al. 2017 |
| *Ceratophyllum demersum* | 3 | 0 | 8 | Yang et al. 2020 |
| *Chenopodium quinoa* | 1 | 1 | 6 | Zou et al. 2017 |
| *Cinnamomum kanehirae* | 2 | 0 | 4 | Chaw et al. 2019 |
| *Citrus sinensis* | 0 | 1 | 3 | Qiao et al. 2019 |
| *Coffea canephora* | 0 | 1 | 3 | Qiao et al. 2019 |
| *Cucumis sativus* | 0 | 1 | 3 | Qiao et al. 2019 |
| *Datisca glomerata* | 0 | 1 | 3 | Ren et al. 2018 |
| *Daucus carota* | 2 | 1 | 12 | Qiao et al. 2019 |
| *Dianthus caryophyllus* | 0 | 1 | 3 | Qiao et al. 2019 |
| *Dimocarpus longan* | 0 | 1 | 3 | Lin et al. 2017 |
| *Elaeis guineensis* | 2 | 0 | 4 | Qiao et al. 2019 |
| *Eucalyptus grandis* | 1 | 1 | 6 | Qiao et al. 2019 |
| *Euryale ferox* | 1 | 1 | 6 | Yang et al. 2020 |
| *Fraxinus excelsior* | 2 | 1 | 12 | Van de Peer et al. 2017 |
| *Glycine max* | 2 | 1 | 12 | Qiao et al. 2019 |
| *Helianthus annuus* | 1 | 2 | 18 | Badouin et al. 2017 |
| *Humulus lupulus* | 0 | 1 | 3 | Qiao et al. 2019 |
| *Ipomoea trifida* | 0 | 2 | 9 | Li et al. 2019 |
| *Juglans regia* | 1 | 1 | 6 | Qiao et al. 2019 |
| *Kalanchoe fedtschenkoi* | 2 | 1 | 12 | Yang et al. 2017 |
| *Kobresia littledalei* | 2 | 0 | 4 | Can et al. 2020 |
| *Linum usitatissimum* | 1 | 1 | 6 | Qiao et al. 2019 |
| *Liriodendron chinense* | 1 | 0 | 2 | Chen et al. 2019 |
| *Lonicera japonica* | 1 | 1 | 6 | Ren et al. 2018 |
| *Macleaya cordata* | 1 | 0 | 2 | Liu et al. 2017 |
| *Malus domestica* | 1 | 1 | 6 | Qiao et al. 2019 |
| *Manihot esculenta* | 1 | 1 | 6 | Qiao et al. 2019 |
| *Medicago truncatula* | 1 | 1 | 6 | Qiao et al. 2019 |
| *Moringa oleifera* | 1 | 1 | 6 | Qiao et al. 2019 |
| *Morus notabilis* | 0 | 1 | 3 | Qiao et al. 2019 |
| *Musa acuminata* | 4 | 0 | 16 | Qiao et al. 2019 |
| *Nelumbo nucifera* | 1 | 0 | 2 | Qiao et al. 2019 |
| *Panax notoginseng* | 1 | 1 | 6 | Zhang et al. 2017 |
| *Phalaenopsis equestris* | 2 | 0 | 4 | Qiao et al. 2019 |
| *Piper nigrum* | 1 | 0 | 2 | Hu et al. 2019 |
| *Populus trichocarpa* | 1 | 1 | 6 | Qiao et al. 2019 |
| *Primula veris* | 0 | 1 | 3 | Qiao et al. 2019 |
| *Punica granatum* | 1 | 1 | 6 | Qin et al. 2019 |
| *Rhizophora apiculata* | 1 | 1 | 6 | Xu et al. 2017 |
| *Sesamum indicum* | 1 | 1 | 6 | Qiao et al. 2019 |
| *Solanum lycopersicum* | 0 | 2 | 9 | Qiao et al. 2019 |
| *Spirodela polyrhiza* | 2 | 0 | 4 | Qiao et al. 2019 |
| *Tarenaya hassleriana* | 1 | 2 | 18 | Qiao et al. 2019 |
| *Theobroma cacao* | 0 | 1 | 3 | Qiao et al. 2019 |
| *Tripterygium wilfordii* | 0 | 2 | 9 | Tu et al. 2020 |
| *Utricularia gibba* | 3 | 1 | 24 | Qiao et al. 2019 |
| *Vitis vinifera* | 0 | 1 | 3 | Qiao et al. 2019 |
| *Ziziphus jujuba* | 0 | 1 | 3 | Qiao et al. 2019 |
| *Zostera marina* | 1 | 0 | 2 | Qiao et al. 2019 |
| *Zea mays* | 4 | 0 | 16 | Qiao et al. 2019 |

**Table S4.** The output of two linear regressions with divergence time as predictors of LTRs insertion time and obtained residuals as predictors of genome size, respectively.

| **Family age (n=73): R^2^=0.001392** | | | | |
| --- | --- | --- | --- | --- |
|  | Estimate | Std. Error | t value | p-value |
| Intercept | 2.340812 | 0.295353 | 7.925 | 2.3e-11 |
| Family age (Mya) | 0.001129 | 0.003587 | 0.315 | 0.754 |
| **Insertion time (n=73): R^2^=0.056** | | | | |
|  | Estimate | Std. Error | t value | p-value |
| Intercept | 0.408038 | 0.038444 | 10.614 | 2.66e-16 |
| Insertion time (Mya) | -0.085811 | 0.041691 | -2.058 | 0.0432 |

**Table S5.** Regression output testing association between a series of correlation factors and genome size fold (genome size scaled by ancestral genome size of angiosperms).

| **LTRs:** | | | | | | | | |
| --- | --- | --- | --- | --- | --- | --- | --- | --- |
|  | | | Linear regression | | | PGLS model | | |
|  | Estimate | Std. Error | t value | p-value | Estimate | Std. Error | t value | p-value |
| Intercept | -0.162795 | 0.055386 | -2.939 | 0.0044 | -0.153306 | 0.291129 | -0.527 | 0.6001 |
| LTR (%) | 0.016192 | 0.001413 | 11.463 | 0.0000 | 0.016387 | 0.001446 | 11.334 | 0.0000 |
| **LINEs:** | | | | | | | | |
|  | | | Linear regression | | | PGLS model | | |
|  | Estimate | Std. Error | t value | p-value | Estimate | Std. Error | t value | p-value |
| Intercept | 0.404488 | 0.052660 | 7.681 | 6.03e-11 | 0.406963 | 0.483386 | 0.842 | 0.4027 |
| LINE (%) | 0.002013 | 0.008580 | 0.235 | 0.815 | 0.005611 | 0.010988 | 0.511 | 0.6112 |
| **SINEs:** | | | | | | | | |
|  | | | Linear regression | | | PGLS model | | |
|  | Estimate | Std. Error | t value | p-value | Estimate | Std. Error | t value | p-value |
| Intercept | 0.414183 | 0.059729 | 6.934 | 1.46e-09 | 0.450920 | 0.481224 | 0.937 | 0.3519 |
| SINE (%) | -0.006517 | 0.199148 | -0.033 | 0.974 | -0.066892 | 0.225895 | -0.296 | 0.7860 |
| **DNA transposons:** | | | | | | | | |
|  | | | Linear regression | | | PGLS model | | |
|  | Estimate | Std. Error | t value | p-value | Estimate | Std. Error | t value | p-value |
| Intercept | 0.432323 | 0.080961 | 5.340 | 1.04e-06 | 0.561761 | 0.488121 | 1.151 | 0.2536 |
| DNA transposons (%) | -0.002367 | 0.008544 | -0.277 | 0.783 | -0.011443 | 0.010205 | -1.121 | 0.2659 |
| **Tandem repeats:** | | | | | | | | |
|  | | | Linear regression | | | PGLS model | | |
|  | Estimate | Std. Error | t value | p-value | Estimate | Std. Error | t value | p-value |
| Intercept | 0.423678 | 0.059905 | 7.072 | 8.12e-10 | 0.478848 | 0.481684 | 0.994 | 0.3235 |
| Tandem repeats (%) | 0.002465 | 0.010165 | -0.243 | 0.809 | -0.008091 | 0.011623 | -0.696 | 0.4886 |
| **Transoposable Elements:** | | | | | | | | |
|  | | | Linear regression | | | PGLS model | | |
|  | Estimate | Std. Error | t value | p-value | Estimate | Std. Error | t value | p-value |
| Intercept | -0.231383 | 0.075983 | -3.045 | 3.25e-03 | -0.332333 | 0.328156 | -1.012 | 0.3146 |
| TE (%) | 0.013380 | 0.001476 | 9.062 | 1.6e-13 | 0.014608 | 0.001533 | 9.528 | 0.0000 |
| **Polyploidization Fold:** | | | | | | | | |
|  | | | Linear regression | | | PGLS model | | |
|  | Estimate | Std. Error | t value | p-value | Estimate | Std. Error | t value | p-value |
| Intercept | 0.376166 | 0.078245 | 4.808 | 1.12e-05 | 0.458447 | 0.516039 | 0.888 | 0.3780 |
| PF (%) | 0.007208 | 0.010029 | 0.719 | 0.475 | -0.000802 | 0.010139 | -0.079 | 0.9373 |
| **Insertion time (Mya):** | | | | | | | | |
|  | | | Linear regression | | | PGLS model | | |
|  | Estimate | Std. Error | t value | p-value | Estimate | Std. Error | t value | p-value |
| Intercept | 0.408038 | 0.038444 | 10.61 | 2.66e-16 | 0.629297 | 0.483687 | 1.301 | 0.1975 |
| Insertion time | 0.085811 | 0.041691 | -2.058 | 0.0432 | -0.072419 | 0.042709 | -1.696 | 0.0943 |

**Reference**

Badouin H, Gouzy J, Grassa CJ, Murat F, Staton SE, et al. 2017. The sunflower genome provides insights into oil metabolism, flowering and Asterid evolution. Nature 546(7656):148-152.

Can M, Wei W, Zi H, Bai M, Liu Y, et al. 2020. Genome sequence of Kobresia littledalei, the first chromosome-level genome in the family Cyperaceae. Sci Data 7, 175.

Chaw SM, Liu YC, Wu YW, Wang HY, Lin CI, et al. 2019. Stout camphor tree genome fills gaps in understanding of flowering plant genome evolution. Nat Plants Jan;5(1):63-73.

Chen J, Hao Z, Guang X, Zhao C, Wang P, et al. 2019. Liriodendron genome sheds light on angiosperm phylogeny and species–pair differentiation. Nat Plant 5, 18-25.

Fukushima K, Fang X, Alvarez-Ponce D, Cai H, Carretero-Paulet L, et al. 2017. Genome of the pitcher plant Cephalotus reveals genetic changes associated with carnivory. Nat Ecol Evol 1(3):59.

Harkess A, Zhou J, Xu C, Bowers JE, Van der Hulst R, et al. 2017. The asparagus genome sheds light on the origin and evolution of a young Y chromosome. Nat Commun 8(1):1279.

Hu L, Xu Z, Wang M, Fan R, Yuan D, et al. 2019. The chromosome-scale reference genome of black pepper provides insight into piperine biosynthesis. Nat Commun 10, 4702.

Li M, Yang S, Xu W, Pu Z, Feng J, et al. 2019. The wild sweetpotato (Ipomoea trifida) genome provides insights into storage root development. BMC Plant Biology 19(1):119.

Lin YL, Min JM, Lai RL, Wu ZY, Chen YK, et al. 2017. Genome-wide sequencing of longan (Dimocarpus longan Lour.) provides insights into molecular basis of its polyphenol-rich characteristics. Gigascience 6(5):1-14.

Liu X, Liu Y, Huang P, Ma Y, Qing Z, et al. 2017. The Genome of Medicinal Plant Macleaya cordata Provides New Insights into Benzylisoquinoline Alkaloids Metabolism. Mol Plant 10(7):975-989.

Qiao X, Li Q, Yin H, Qi K, Li L, et al. 2019. Gene duplication and evolution in recurring polyploidization–diploidization cycles in plants. Genome Biology 20:38.

Qin G, Xu C, Ming R, Tang H, Guyot R, et al. 2019. The pomegranate (Punica granatum L.) genome and the genomics of punicalagin biosynthesis. Plant J 91(6):1108-1128.

Ren R, Wang H, Guo C, Zhang N, Zeng L, et al. 2018. Wide-Spread Whole Genome Duplications Contribute to Genome Complexity and Species Diversity in Angiosperms. Mol. Plant 11(3):414-428.

Salojärvi J, Smolander OP, Nieminen K, Rajaraman S, Safronov O, et al. 2017. Genome sequencing and population genomic analyses provide insights into the adaptive landscape of silver birch. Nat Genet 49(6):904-912.

Tu L, Su P, Zhang Z, Gao L, Wang J, et al. 2020. Genome of Tripterygium wilfordii and identification of cytochrome P450 involved in triptolide biosynthesis. Nat Commun 11(1):971.

Van de Peer Y, Mizrachi E, Marchal K. 2017. The evolutionary significance of polyploidy. Nat Rev Genet 18(7):411-424.

Wei C, Yang H, Wang S, Zhao J, Liu C, et al. 2018. Draft genome sequence of Camellia sinensis var. sinensis provides insights into the evolution of the tea genome and tea quality. PNAS 115(18): E4151-E4158.

Xu S, He Z, Zhang Z, Guo W, Lyu H, et al. 2017. The origin, diversification and adaptation of a major mangrove clade (Rhizophoreae) revealed by whole-genome sequencing. Natl Sci Rev 4(5):721-734.

Yang X, Hu R, Yin H, Jenkins J, Shu S, et al. 2017. The Kalanchoë genome provides insights into convergent evolution and building blocks of crassulacean acid metabolism. Nat Commun 8(1):1899.

Yang Y, Sun P, Lv L, Wang D, Ru D, et al. 2020. Prickly waterlily and rigid hornwort genomes shed light on early angiosperm evolution. Nat Plants 10.1038/s41477-020-0594-6.

Zhang D, Li Wei, Xia EH, Zhang QJ, Yuan Liu, et al. 2017. The Medicinal Herb Panax notoginseng Genome Provides Insights into Ginsenoside Biosynthesis and Genome Evolution. Mol. Plant 10(6):903-90.

Zou C, Chen A, Xiao L, Muller HM, Ache P, et al. 2017. A high-quality genome assembly of quinoa provides insights into the molecular basis of salt bladder-based salinity tolerance and the exceptional nutritional value. Cell Res 27(11):1327-1340.
